# Global public health intelligence: World Health Organization operational practices

**DOI:** 10.1371/journal.pgph.0002359

**Published:** 2023-09-20

**Authors:** Esther Hamblion, Neil J. Saad, Blanche Greene-Cramer, Adedoyin Awofisayo-Okuyelu, Dubravka Selenic Minet, Anastasia Smirnova, Etsub Engedashet Tahelew, Kaja Kaasik-Aaslav, Lidia Alexandrova Ezerska, Harsh Lata, Sophie Allain Ioos, Emilie Peron, Philip Abdelmalik, Enrique Perez-Gutierrez, Maria Almiron, Masaya Kato, Amarnath Babu, Tamano Matsui, Viema Biaukula, Pierre Nabeth, Aura Corpuz, Jukka Pukkila, Ka-Yeung Cheng, Benido Impouma, Etien Koua, Abdi Mahamud, Phillipe Barboza, Ibrahima Socé Fall, Oliver Morgan, World Health Organization Public Health Intelligence teams

**Affiliations:** 1 Department of Alert and Response Coordination, Health Emergencies Programme, World Health Organization, Geneva, Switzerland; 2 Epidemic and Pandemic Preparedness and Prevention Department, Health Emergencies Programme, World Health Organization, Geneva, Switzerland; 3 WHO Hub for Pandemic and Epidemic Intelligence, Health Emergencies Programme, World Health Organization, Berlin, Germany; 4 Health Emergency Information & Risk Assessment, Health Emergencies, World Health Organization Regional Office for the Americas, Washington DC, United States of America; 5 Health Emergencies Programme, World Health Organization South-East Asia Regional Office, New Delhi, India; 6 Health Emergencies Programme, World Health Organization Western Pacific Regional Office, Manilla, Philippines; 7 Health Emergencies Programme, World Health Organization Eastern Mediterranean Regional Office, Cairo, Egypt; 8 Health Emergencies Programme, World Health Organization European Regional Office, Copenhagen, Denmark; 9 Health Emergencies Programme, World Health Organization Africa Regional Office, Brazzaville, Congo; 10 Office of the Assistant Director-General for Emergencies Response, Health Emergencies Programme, World Health Organization, Geneva, Switzerland; 11 Department of Health Emergency Interventions, Health Emergencies Programme, World Health Organization, Geneva, Switzerland; George Mason University School of Public Policy, UNITED STATES

## Abstract

Early warning and response are key to tackle emerging and acute public health risks globally. Therefore, the World Health Organization (WHO) has implemented a robust approach to public health intelligence (PHI) for the global detection, verification and risk assessment of acute public health threats. WHO’s PHI operations are underpinned by the International Health Regulations (2005), which require that countries strengthen surveillance efforts, and assess, notify and verify events that may constitute a public health emergency of international concern (PHEIC). PHI activities at WHO are conducted systematically at WHO’s headquarters and all six regional offices continuously, throughout every day of the year. We describe four interlinked steps; detection, verification, risk assessment, and reporting and dissemination. For PHI operations, a diverse and interdisciplinary workforce is needed. Overall, PHI is a key feature of the global health architecture and will only become more prominent as the world faces increasing public health threats.

## Introduction

Early detection and rapid response are essential to tackle public health threats. They save lives, reduce negative public health impact and safeguard wellbeing [1–4]. Public health threats occur daily and have the potential to occur more frequently due to a multitude of factors, including climate change, urbanisation and global population movement [5–7]. Unchecked or tardily detected, public health emergencies can have disastrous consequences for individuals and communities [1,2,8].

The World Health Organization (WHO) occupies a unique position in the global health landscape. It is the sole organisation that has the legal mandate to safeguard health globally, a far-reaching organizational structure of regional and country offices, the authority to interact with WHO Member States, as well as an extensive network of global and local health partners. One of WHO’s most important activities is the detection of and response to acute public health events. In 1997, WHO first established a dedicated global disease surveillance system to respond to (re-)emerging and novel threats [9,10]. This system was underpinned by the International Health Regulations (2005) (IHR (2005)), a legally binding agreement, which came into force in June 2007 [11]. Under the IHR (2005), State Parties, which include all 194 Member States, the Principality of Liechtenstein, and the Holy See, agreed to strengthen surveillance efforts, and assess, notify and verify events that may constitute a public health emergency of international concern (PHEIC) [11]. In 2016, during the 69^th^ World Health Assembly, WHO’s mandate was strengthened further by the establishment of the WHO Health Emergencies Programme following the Organization’s delayed detection, response and communication on the 2014–16 Ebola outbreak in West Africa [12,13]. WHO has implemented and continuously furthers its robust approach to public health intelligence (PHI) for the global detection, verification and risk assessment of acute public health threats. Overall, PHI is a core public health function for detecting, verifying, analysing, assessing, interpreting and generating information for action and dissemination [14]. WHO’s approach to PHI combines multiple sources of data with detailed on-the-ground verification and local contextual information [15–17]. A key purpose of PHI is to detect and verify public health risks as early as possible in order to mitigate and reduce the impact among individuals and populations [17–19]. This goal, in particular, can make the importance of PHI difficult for policy-makers to evaluate as success is not readily enumerated in cases or deaths averted. The operational practice of conducting global PHI activities at WHO has developed considerably over recent years, with dedicated teams based at headquarters and in the six regional offices. However, these developments have not previously been widely communicated. Describing WHO’s general operational practices will increase transparency about how WHO conducts PHI, will allow others to better understand, adapt, and improve their own PHI operations, as well as enable identification of areas of potential collaboration between WHO and other organizations.

## Conducting public health intelligence operations

Operationalisation of PHI activities across WHO globally is underpinned by the legal mandate derived from the IHR (2005). The revised IHR (2005) was developed in response to increasing global health threats and as a result of lessons learnt, in particular related to detection, verification and information sharing, during the response to outbreaks, such as the 2003 severe acute respiratory syndrome (SARS) outbreak [20–22].

**Fig 1:**
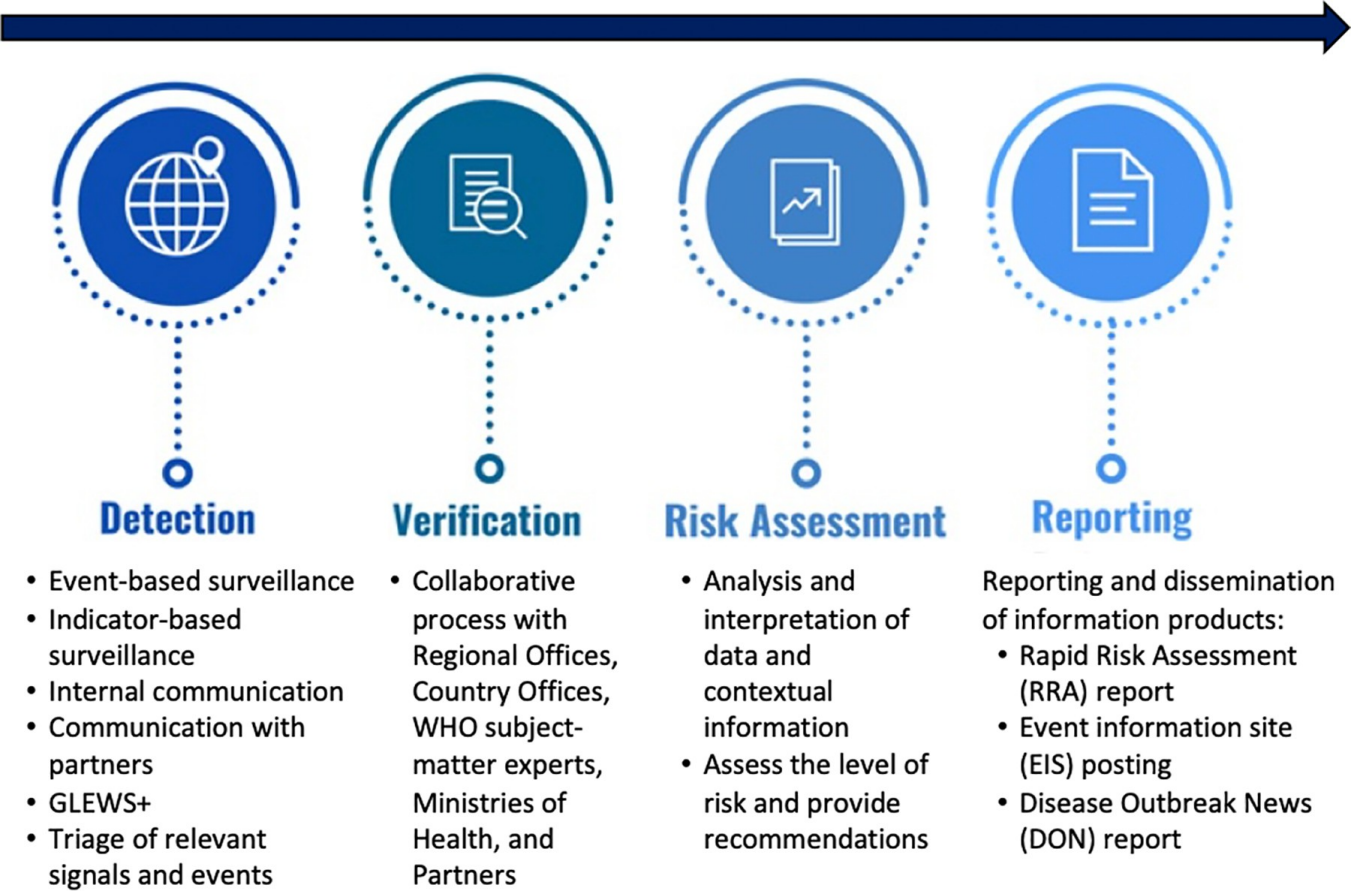
Framework of public health intelligence activities at WHO.

PHI activities at WHO are conducted continuously throughout every day of the year to systematically detect, verify and respond to acute public health threats around the world. Specifically, it involves four interlinked steps to guide public health action and decision-making: detection, verification, risk assessment, and reporting and dissemination (**Fig 1**). Briefly, a team continuously scans a wide range of information sources (see below) for signals of potential public health events [15,16]. Verification of the signals involves working closely with national authorities in collaboration with WHO Regional Office and Country Office staff; signals that are verified are termed “events” [15,16]. A risk assessment for events is then conducted (further details below) following which several information products are generated to guide decision-makers, principally WHO and Member States, and facilitate response activities [23]. In subsequent Sections, each of the steps is discussed in depth.

### Detecting public health threats

WHO has an all-hazards approach to signal detection, which means that threats of any nature–including infectious diseases but also natural disasters, geohazards and human-made conflicts that can cause public health events of international concern–are assessed. Signals of potential public health threats include any indication of an usual event, such as the occurrence of elevated morbidity or mortality in animals or humans, in a given geographical area and in a specific timeframe, an unexpected cluster of cases or deaths, (re-)emergence of an infectious disease, and health threats with the potential of causing substantial morbidity or mortality (full overview of criteria provided in **S1 Table)** [15,16].

**Fig 2:**
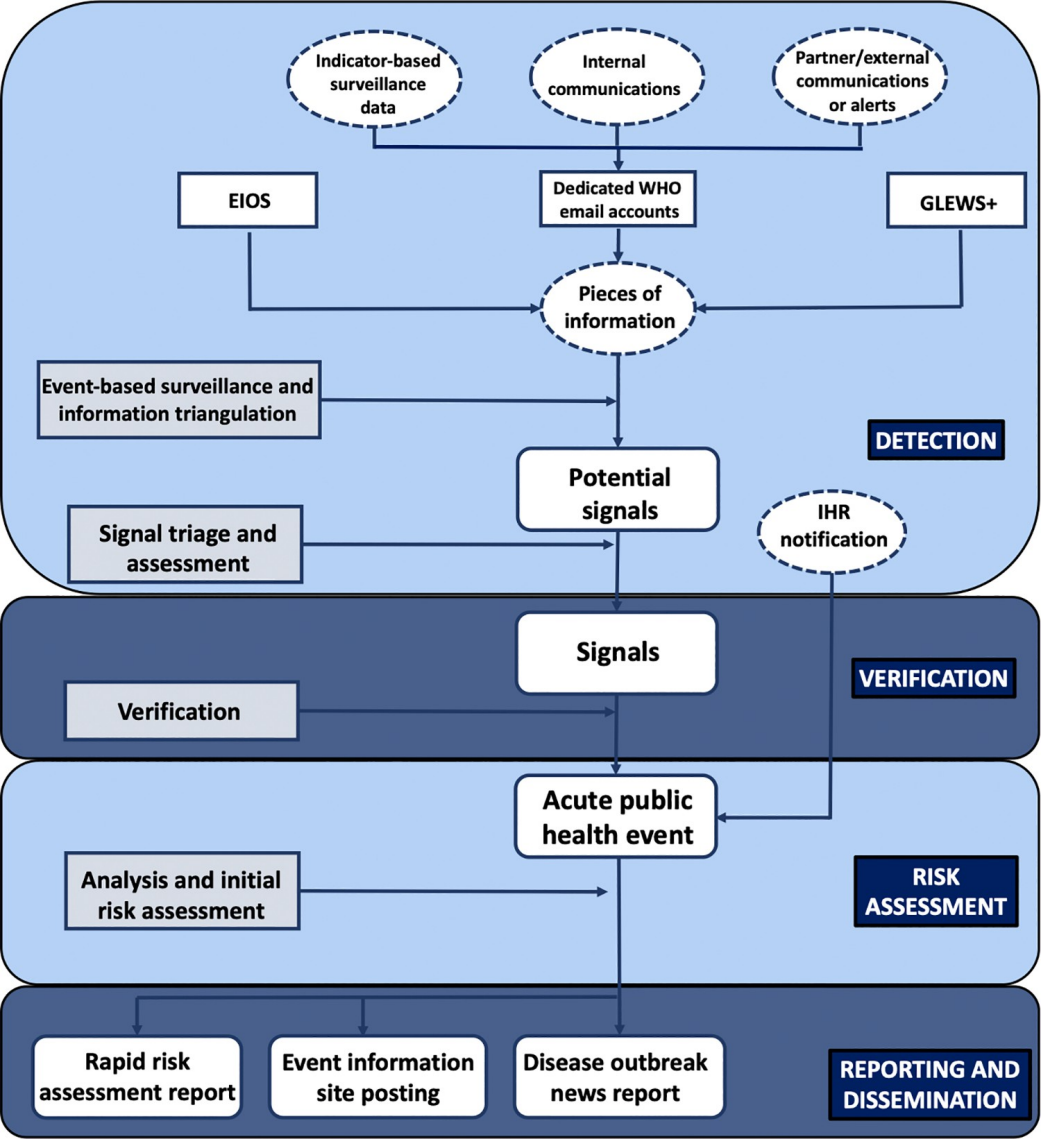
World Health Organization public health intelligence information flow, associated outputs, and activities.

To capture signals, WHO uses a variety of information channels (**Fig 2**). One of the main channels currently used is the Epidemic Intelligence from Open Sources (EIOS) system, which processes information from online news and social media, government and official websites, and blogs and expert groups, and applies natural language processing tools that facilitate screening of information for signals of interest [24–26]. A second information channel is facilitated by dedicated WHO email accounts, for direct communications, including information alerts (from, for example, the Global Disaster and Alert Coordination System [27]) and information sharing from partners and WHO colleagues, such as field investigation reports or data, including from indicator-based surveillance. A third information channel is GLEWS+, a global early warning system for health threats and emerging risks at the human-animal interface, maintained jointly by the Food and Agriculture Organization, WHO, and the World Organisation for Animal Health [28].

EIOS: Epidemic Intelligence from Open Sources system; GLEWS+: a global early warning system for health threats and emerging risks at the human-animal interface, maintained jointly by the Food and Agriculture Organization, WHO, and the World Organization for Animal Health. Not all acute public events will have a Rapid risk assessment report, Event information site posting, or Disease outbreak news report. Rapid risk assessment reports are internal to the WHO and the wider United Nations. Event information site postings are shared with WHO Member States via their NFPs, authorized WHO staff and selected international governmental organizations. DON reports are shared with the public through the WHO website.

Pieces of information received through the main information channels can, for example through EIOS, include a media report on unexpected deaths or an elevated number of cases. Alternatively, it could be a situation or field investigation report by a local or international NGO, governmental surveillance update, an email alert on natural disasters and weather patterns from the Global Disaster and Alert Coordination System, or a communication on a disease outbreak in animals with zoonotic potential from a GLEWS+ partner, which are shared through the dedicated WHO email account.

Potential signals undergo triaging by triage analysts and detailed signal assessment to determine whether they are true signals or not. This entails triangulating the information from the potential signal with other contemporaneous reports, reviewing the information against previous trends or occurrences, contextualising the situation, and discussions with subject-matter and disease experts. After signal triaging and assessment according to predefined criteria (**S1 Table**), potential signals can be (1) discarded as not being a relevant public health threat, (2) be retained for further close monitoring, or (3) be designated as a true signal and a request for further information or verification under the IHR (2005) can be sent. Close monitoring of potential signals is generally conducted for potential signals that are of concern but do not meet the threshold to be considered a signal. Close monitoring will occur in the days following the initial detection and, depending on the type of disease, condition, or hazard, can be up for a period of up to one or two weeks. If the information in this period does not result in the potential signal meeting the threshold to be considered as a signal, then it is discarded. However, subject-matter and disease experts at WHO routinely monitor and stay abreast of the latest trends in their area of expertise, with whom information on potential signals can be shared to be included in routine periodic monitoring.

In addition to events being detected by WHO they may instead be directly reported to WHO by the National IHR Focal point (NFP) using the decision instrument of Annex 2 of the IHR (2005). When NFPs report public health events of concern these are considered by WHO as events directly (**Fig 2**). Countries may also liaise with WHO directly for consultation on potential acute public health events. In addition, WHO also has the ability and capacity, through the extensive local presence and networks, to verify signals and report on events when IHR verification from Member States is pending.

### Verifying public health threats

A vital and unique feature of verification at WHO is the local presence of WHO in 147 countries, territories and areas, the support given to national authorities with the verification, and ability to provide detailed contextual understanding of the situation.

To assess the authenticity of a signal a request for further information or confirmation is sent to national authorities but can also be sent to WHO regional or country offices or to other UN agencies, partners or technical networks present in the country. However, the veracity of a signal may be officially ascertained by sending a formal verification request to the NFP, as designated by the Member State, which are a national office or centre that is continuously available for IHR-related communications with WHO [11]. Following a confirmatory response, to the IHR verification request, a signal can be designated as an event as defined under the IHR (2005). Alternatively, if the information provided through the requests negates any of the criteria that led to the signal being selected or if it does not meet the criteria to be designated an event, then the signal can be either discarded or retained for active monitoring.

### Assessing public health threats: Analysis and risk assessment

All events undergo analysis and an initial risk assessment (**Fig 2**). This risk assessment evaluates public health threats against the following criteria: the potential public health impact, risk of international spread, risk of trade or travel restrictions, and if the event is considered epidemiologically unusual. Moreover, the initial risk assessment is repeated every time new information about the event is available to continuously monitor changes in the risk.

Some events will undergo a further more in-depth rapid risk assessment (RRA), which is conducted by WHO independently, using a published methodology [23]. Criteria for initiating an RRA include events requiring WHO support to respond, events of pathogens of epidemic potential (high morbidity/mortality or rapid spread), events with unknown aetiology, and events involving multiple countries (Further details in **S2 Table**). RRAs aim to assess the risk of the event to adversely impact public health and will evaluate the need for WHO follow-up actions, including communication about the risks. A RRA will assess the hazard, exposure and context. It determines the levels of risk (at national, regional and global level) as well as an overall public health risk based on three risk questions, which include the potential risk to human health, risk of spread, and risk of insufficient control capacity, with additional risk questions added if relevant to the event. The risk for each question is based on the likelihood and the severity of potential consequences, with a justification included for why they were considered as such [23]. RRAs are disseminated within WHO, the wider United Nations, and partners through the Global Outbreak Alert and Response Network (GOARN) to inform and support decision making as well as facilitate appropriate and timely mitigation and control measures.

### Reporting and information dissemination

Information dissemination is key for effective rapid response to acute public health events. WHO produces two key information products related to public health intelligence activities, which are mandated under the IHR (2005); a posting on the Event Information Site for IHR national focal points (EIS) and disease outbreak news (DON) reports for the public (**Fig 2**). WHO has a unique role in communication with Member States for which it uses the EIS platform. This is a closed WHO platform to which only NFPs, authorised WHO staff and selected international governmental organizations have access, and on which EIS postings are published. The EIS platform has been in use since 2007, when IHR (2005) came into force, and is used to inform Member States on acute public health events with accurate information, including a WHO risk assessment. Through EIS, WHO shares information, in confidence, about acute public health events which allow Member States to prepare and enhance response efforts. The EIS posting includes the assessment of the event based on the risk questions from Annex 2 of the IHR (2005) (unexpected or unusual event, serious public health impact, disease spread, and restriction on travel or trade) as well as a short description of the public health event, a summary of the WHO risk assessment, a section on response, and WHO advice to Member States [29,30].

For communicating to the public, WHO employs a wide variety of approaches, including press conferences, social media, dashboards, bulletins, and region- or country-specific (situation) reports. In addition, there are DON reports, which provide reliable information on acute public health events to the public and have been produced since 1996. DON reports are published on the WHO website and are among the most visited WHO webpages. DON reports include a summary of the public health event, the ongoing public health response, a summary of the WHO risk assessment and a section on WHO advice [31]. DON reports are a highly valuable source of information on ongoing acute public health events but the format has changed considerably since they were first published in 1996. The first DON report consisted of a single paragraph and this has now grown into a report with several sections that provide detailed information. The layout and structure of DON reports is regularly reviewed with the aim to improve information sharing and increase robustness of DON reporting.

With protracted public health events, DON reports will be initially produced but can transition into regular (generally weekly or fortnightly) situation reports, as with COVID-19 and the multi-country outbreak of mpox [32,33]. Also WHO Regions will communicate on new or ongoing acute public health events through weekly reports and bulletins [34,35]. In addition, WHO communicates with the global public health community on the latest evidence and findings from outbreak investigations or public health activities through scientific publications [36–38].

## Managing public health intelligence operations

PHI operations at WHO are conducted by dedicated WHO staff, who work with technical and subject matter experts, when necessary. At WHO there are 12 key roles across six areas of activity needed for PHI operations (**Table 1**). Four of the six areas of activity align with the four steps in PHI and include detection, verification, risk assessment and information dissemination, with several roles in each of these areas of activity. In addition, there are two further essential areas of activity: (1) leadership and coordination, which is necessary for the overall strategic vision and operational management, as well as (2) operational support, which encompasses roles that assist the operationalisation and improvement of PHI operations. Training and experience in public health and epidemiology is required for all roles but each role in the various activity areas has additional specific skills that are required. In addition, diversity in terms of background, culture, language, experience, and expertise is what makes an ideal PHI team. Importantly, depending on the scope of PHI activities, multiple of the described roles could be filled by a single individual or, alternatively, multiple individuals might be required for a single role. Moreover, the number of individuals required will depend on (1) the scope of PHI activities, which could be global, national or subnational; (2) focus, which could be all-hazards or a set of specific public health threats; and (3) timeliness, in which threats should be detected and information disseminated. At WHO, the aforementioned roles exist in the dedicated PHI teams at headquarters and in the six Regional Offices while some of the roles will be conducted by WHO staff in Country Offices, as part of their involvement in the verification and risk assessment activities. A general example of how PHI operations occur in practice at WHO is given in Box 1 to facilitate the contextualisation.

**Box 1. Public health intelligence activities in practice: An example**

As part of the detection activities on a weekday, the event-based surveillance (EBS) analyst at WHO headquarters detected a media article using EIOS. The media article mentions an unexpected number of deaths and elevated number of cases who are experiencing fever, vomiting and diarrhoea in the northern province of country X. The EBS analyst suspects this might be an outbreak of cholera and will triangulate the information in the article with information from other sources, if possible, and compare with data on historical trends in the country and/or area. After triangulating and further assessment, the EBS analyst finds that the reported number of deaths and cases are unusual and unexpected. They find that cholera generally does not occur in that part of the country and that the case fatality ratio (CFR) is rather elevated for a potential cholera outbreak. After discussions with the cholera and diarrhoeal disease experts at WHO, they decide that this potential signal meets the criteria to be considered a signal. They share the information on the signal with the WHO Country Office, through the WHO Regional Office, for verification of the information. The next day the information from the media report is confirmed by the WHO Country Office, after discussing with Ministry of Health counterparts. The National IHR Focal point (NFP) also notifies WHO under IHR (2005) and shares updated information, which showed that the cholera outbreak is spreading. After the initial risk assessment is conducted, the WHO headquarters team decides that the increasing spread and high CFR warrants an in-depth risk assessment, the rapid risk assessment (RRA). This latter risk assessment is conducted by the risk assessment analyst in collaboration with WHO Regional and Country Office colleagues to guide WHO decision-making and response activities. As part of the RRA process, which is conducted in 2–3 days after the media report was verified, it is decided that WHO Member states should be formally informed through a posting on the Event Information Site for IHR national focal points (EIS) as the event meets at least of the two criteria in Annex 2 of the IHR (2005). In addition, as further reporting shows that the number of cases and deaths continue to increase, WHO colleagues agree that the public should be provided with verified and authoritative information on the outbreak through a disease outbreak news (DON) report, which will be drafted and coordinated by the information dissemination analyst.

**Table 1:** Roles necessary for conducting public health intelligence, by activity.

Activity	Role(s)	Description	Specific skills*
Leadership and coordination	Strategic lead	Overall leadership and strategic coordination of public health intelligence (PHI) activities.	Leadership, strategic vision, team management
	Operational lead	Leadership and coordination of PHI activities within area of focus.	Coordination, people management, topic expert
Detection	Event-based surveillance analyst	Analyst in charge of routine event-based surveillance monitoring of open source information.	Disease and topic expert, disease surveillance, (health) information systems
	Triage analyst	Analyst in charge of (1) monitoring information received through generic email accounts, including indicator-based surveillance information, and (2) managing process and product related communications.	Multi-tasking, diplomatic, communication, priority-setting
	Out-of-hours analyst	Performs triage analyst functions out-of-hours (night and weekends) as well risk assessment analyst functions when time-sensitive risk assessment associated products needed.	Multi-tasking, diplomatic, communication, priority-setting, disease and topic expert, report writing, communication
Verification	Verification teams	Person(s) with understanding of local context who verify potential events on the ground.	Field experience, disease and topic expert, clinical or paramedical
Risk assessment	Risk assessment analyst	Analysis of information from verification teams, conducts and coordinates risk assessment with subject matter experts, and development of associated information products.	Disease and topic expert, field experience, risk assessment, report writing, risk communication
Information dissemination	Information dissemination analyst	Analyst in charge of developing information products, generally for public dissemination.	Report writing, communication, work under pressure
Operational support	Data scientist	Responsible for data cleaning and curation, dashboard development and data visualisations.	Data management, wrangling & visualisation, statistical analysis
	System developer	Interfaces between PHI teams and information technology teams to improve PHI systems for signal detection and event management.	Disease surveillance, process management, health systems, communication
	Monitoring and evaluation analyst	Analyst responsible for analysis of and reporting on end-to-end process of PHI activities, with the aim of continuously optimising PHI operations.	Data management, epidemiological analysis, report writing, communication
	Support assistant	Provides administrative support to strategic and operational leads. Responsible for team human resources and budgetary procedures.	Budget and human resource expertise, communication, coordination, diplomatic

**Abbreviations**: Public health intelligence (PHI). **Note**: Depending on the scope of the public health intelligence activities, multiple of the described roles could be filled by a single individual or, alternatively, multiple individuals might be required for a single role. *The specific skills are in addition to training and experience in public health and epidemiology, which is required for each role.

## Furthering public health intelligence operations

Conducting PHI is well-established at various organisations and at WHO. However, as health threats and their drivers change or novel threats emerge, PHI must continuously improve to remain at the forefront of early detection and response.

Three areas are key for future improvements in PHI operations. First, the growing volume and varied format of data and information complicates data aggregation, curation and screening. In addition, increasing questions about the ethics of large-scale data collection, sharing, protection, and privacy need to be taken into account. Some of these issues might be resolved through modernizing data systems, including those for the screening of open-source information but also those for signal and event management. Moreover, better data linkage, with previous signals and events, as well as population data and geospatial information would greatly facilitate detection and response activities. Additionally, the terminology used in PHI is increasingly blurred and an updated set of definitions that more appropriately reflect contemporary information flows is needed. To tackle these challenges, WHO is currently updating the internal information management systems for signal management and tracking, event management, and communication with NFPs. The updated systems, once in place, will facilitate information flow and data linkage along various PHI activities as well as allow for the integration of novel data sources for signal and event (risk) assessment. Moreover, improved information management will enhance monitoring and evaluation activities, which, in turn, strengthen future operational response and enable sharing of operational findings with the wider global public health community. In addition, WHO are reviewing and updating their internal standard operating procedures and the publicly available guidelines to incorporate the latest practices and facilitate implementation of PHI activities by other global public health actors.

Second, PHI operations are built on collaboration, trust and communication. Collaboration and local connections to ensure active engagement, enhanced information sharing, and rapid verification of acute public health events are essential. Active outreach and continuous relation building should take place to ensure improved understanding and long-term trust. To strengthen relations with NFPs, WHO conducts regular NFP workshops, provides EIOS training to WHO Member States, and continuously develops NFP training materials.

Third, a diverse, multi-cultural, multi-faceted and highly skilled team is necessary for PHI operations as it involves a substantial amount of assessment, analysis and evaluation across a broad spectrum of hazards and diversity of settings. Decision-making across PHI operations whilst under considerable time pressure requires team members with substantial experience, a high level of interpersonal skills as well as sufficient technical resources. Important is the inclusion of social and behavioural and animal or one health expertise, in addition to infectious disease, subject-matter, disease surveillance, risk assessment, and field epidemiology expertise, to the core knowledge in public health and epidemiology of all PHI members. Moreover, there can be a tendency to ignore or minimise the contribution of people in an operational support role, which are essential to facilitate operations. Gaps in those roles might not be evident in the short-term but will undoubtable be noticeably long-term, impact sustainability of operations and increase staff turn-over. Finally, organizations should be a mirror of the people and communities that they aim to serve. A skilled and diverse workforce facilitates insight and allows for more holistic decision-making. WHO is committed to recruiting a diverse, multicultural and gender balanced workforce which includes individuals from across the globe. This directly aligns with what is needed for effective PHI operations and, to facilitate this, WHO has put in place several human resource and talent management policies to support this endeavour.

In summary, PHI is a key component of the global health architecture. The implementation and integration of PHI across various entities, including WHO, has vastly improved detection, risk assessment, and response capabilities. In the years ahead, with novel, (re)-emerging and recurring health threats and the worsening effects of the climate crisis, PHI will prove to be even more impactful. WHO will remain at the forefront and continuously strive to better PHI operations, promote best practices and disseminate evidence to benefit people and populations everywhere.

## Supporting information

S1 Table World Health Organization (WHO) criteria for selecting potential signals. (DOCX)

S2 Table World Health Organization (WHO) criteria for initiating a rapid risk assessment. (DOCX)
