# Supplementary material for: Global public health intelligence: World Health Organization operational practices
Source: PLOS Glob Public Health. 2023 Sep 20;3(9):e0002359. doi: 10.1371/journal.pgph.0002359 (PMC10511126; doi:10.1371/journal.pgph.0002359)
Supplement: S1 Table — (DOCX) [file pgph.0002359.s001.docx]

**S1 Table:** World Health Organization (WHO) criteria for selecting potential signals.

| During the process of event-based surveillance and analysis the triage analyst(s) and event-based surveillance analyst(s) aim to identify potential signals of potential public health concern including human health, animal health (zoonosis or unknown disease with potential threat to human health), food security/food safety, travel and trade, geohazards. | |
| --- | --- |
| In general, the criteria used for the detection of potential signals are: | |
| 1 | Pieces of information reporting the first occurrence, reoccurrence, or unusual or unexpected presentation of a disease/strain in a particular geographic area or population group. |
| 2 | Pieces of information involving a higher than expected mortality or morbidity in humans. |
| 3 | Pieces of information involving an unusual/unexpected/unexplained cluster of disease with similar symptoms (cases or deaths) in the community. |
| 4 | Pieces of information about an occurrence that has the potential to cause an outbreak with a high likelihood of spread. |
| 5 | Pieces of information causing an undue level of concern/fear/panic in the affected community. |
| 6 | Pieces of information regarding an occurrence that may interfere with international trade or travel. |
| 7 | Pieces of information regarding mortality or morbidity among specific population groups (i.e., refugees, health care workers or marginalized populations). |
| 8 | Pieces of information regarding morbidity and/or mortality due to an undiagnosed or unknown disease in a community. |
| 9 | Pieces of information involving unusual/unexpected/unexplained cause of sickness or deaths related to international travel or mass gathering. |
| 10 | Pieces of information regarding unusual mortality and/or morbidity in animal or emerging disease animals with zoonotic potential. |
| 11 | Pieces of information that has potential reputation risk for WHO. |
| 12 | Pieces of information regarding the re-emergence of disease with or without pandemic potential. |
