# Supplementary material for: Global public health intelligence: World Health Organization operational practices
Source: PLOS Glob Public Health. 2023 Sep 20;3(9):e0002359. doi: 10.1371/journal.pgph.0002359 (PMC10511126; doi:10.1371/journal.pgph.0002359)
Supplement: S2 Table — (DOCX) [file pgph.0002359.s002.docx]

**S2 Table**: World Health Organization (WHO) criteria for initiating a rapid risk assessment

| A rapid risk assessment should be systematically considered for all events meeting criteria for notification in the International Health Regulations (2005) Annex 2. In general, the following criteria are taken into account when deciding to conduct a rapid risk assessment [1]: | |
| --- | --- |
| 1 | Events with insufficient information to adequately assess situation with high level of uncertainty. |
| 2 | Event of unknown aetiology. |
| 3 | Events involving multiple countries. |
| 4 | Events with pathogen that has a high potential of spread and/or case fatality. |
| 5 | Events occurring in a vulnerable country, setting or context. For example, a WHO priority country or countries with civil unrest or a weaker health system. |
| 6 | Events that have potential reputation risk for WHO. |
| 7 | Events for which WHO support and or response activities may be anticipated or required. |

**References**

1. World Health Organization. Rapid Risk Assessment of Acute Public Health Events [Internet]. Geneva; 2012. Available: https://www.who.int/publications/i/item/rapid-risk-assessment-of-acute-public-health-events
